# Iron and vitamin D intake on a diet are able to modify the *in vitro* immune response to *Mycobacterium leprae*

**DOI:** 10.1590/0074-02760230178

**Published:** 2024-08-16

**Authors:** Bruna Letícia Martins, Jonatas Perico, Daniele Ferreira de Faria Bertoluci, Adriana Sierra Assencio Almeida Barbosa, Patricia Sammarco Rosa, Maria Renata Sales Nogueira, Vânia Nieto Brito de Souza, Ana Carla Pereira Latini

**Affiliations:** 1 Universidade Estadual Paulista “Julio de Mesquita Filho”, Faculdade de Medicina de Botucatu, Programa de Pós-Graduação em Doenças Tropicais, Botucatu, SP, Brasil; 2 Secretaria de Estado da Saúde, Instituto Lauro de Souza Lima, Governo do Estado de São Paulo, Bauru, SP, Brasil; 3 Universidade Estadual Paulista “Julio de Mesquita Filho”, Faculdade de Medicina de Botucatu, Programa de Pós-Graduação em Patologia, Botucatu, SP, Brasil

**Keywords:** leprosy, vitamin D, iron, Mycobacterium leprae, nutritional status

## Abstract

**BACKGROUND:**

The impact of nutrient availability on the survival of *Mycobacterium leprae* and the development of leprosy remains largely unknown. Iron is essential for the survival and replication of pathogens, while vitamin D has been involved with pathogen elimination and immunoregulation.

**OBJECTIVES:**

We evaluated the influence of dietary iron and vitamin D supplementation and restriction on the inflammatory response of mouse immune cells *in vitro*.

**METHODS:**

After 30 days of standard or modified diets, peritoneal cells and splenocytes were stimulated with the alive microorganisms and sonicated antigens of *M. leprae*, respectively. The production of inflammatory cytokines, reactive oxygen species, and cell proliferation were evaluated.

**FINDINGS:**

In peritoneal cells, vitamin D supplementation and iron restriction reduced the production of IL-6 and TNF in response to *M. leprae*, while splenocytes presented a reduction in TNF production under the same conditions. Lower levels of IFN-γ and TNF were observed in both iron-supplemented and iron-deficient splenocytes. Besides, iron supplementation also reduced the production of IL-6 and IL-10. No changes in the production of reactive oxygen species or in cell proliferation were observed related to different diets.

**MAIN CONCLUSIONS:**

Taken together, these data point to an interference of the status of these nutrients on the interaction between the host and *M. leprae*, with the potential to interfere with the progression of leprosy. Our results highlight the impact of nutritional aspects on this neglected disease, which is significantly associated with unfavourable social conditions.

Leprosy is an infectious disease caused by *Mycobacterium leprae* or *Mycobacterium lepromatosis* with neural and dermatological manifestations.^1^ ^,^ ^2^ The leprosy clinical classification is based on a spectrum with two polar forms, tuberculoid (TT) and lepromatous (LL), and three intermediate forms named borderline-tuberculoid (BT), borderline-borderline (BB), and borderline-lepromatous (BL).^3^ The TT pole is characterised by cellular immunity mediated by T helper cells type 1 (Th1) with the secretion of pro-inflammatory cytokines such as interferon-γ (IFN-γ) and IL-2. The LL pole is characterised by the predominance of the humoral response, mediated by Th2, and the secretion of anti-inflammatory cytokines such as IL-4, and IL-10, which promote a favourable microenvironment for the proliferation of the microorganism and the progression of leprosy.^3^ ^,^ ^4^ ^,^ ^5^ Besides, mainly for treatment purposes, clinical presentations with a low bacillary burden from the tuberculoid pole are classified as paucibacillary (PB), while the opposite pole with a high bacillary burden is classified as multibacillary (MB).^6^

In addition to the classical immune system responses, nutritional immunity mechanisms, which limit the bioavailability of essential nutrients to the pathogen, can be used by the immune system against infections.^7^ Essentially, the restriction of elements involved in the metabolic processes of the pathogen can impact its multiplication and, consequently, the progression of the infectious diseases.^8^ The restriction of transition metals such as iron, zinc, and manganese by the host against the pathogen is the most investigated strategy within nutritional immunity.^9^ ^,^ ^10^ Metals have a prominent position due to their involvement in numerous processes ranging from bacterial metabolism to the regulation of virulence factors.^11^

Most evidence on the role of iron in mycobacterioses derives from tuberculosis (TB). Studies corroborate the influence of iron levels on the progression of TB, as well as excess iron favouring the proliferation of *Mycobacterium tuberculosis (MTB)*.^12^ ^,^ ^13^ The anaemia of chronic disease observed in leprosy patients is a functional deficiency of iron due to its intracellular accumulation, which is mediated by the inflammatory process present, mainly by IL-6.^14^ Oktaria et al.^15^ showed that IL-6 and iron levels are potential markers of the disease progression in individuals living in leprosy endemic areas.

On the other hand, these metals participate in the bactericidal functions of cells of the immune system of the host, such as macrophages, and act as cofactors for biological processes. Therefore, it is essential to highlight that nutrition exerts a significant influence on the immune system, and both deficiencies and excesses of certain micronutrients can impair immunity, affecting the function of immune cells.^9^ ^,^ ^11^ ^,^ ^16^

Other micronutrients acquired through the diet, such as vitamins, fatty acids, and amino acids, also offer support to the immune system, for example, aiding in the biosynthesis of immunoglobulins, cytokines, and receptors. Vitamin D is a nutrient widely related to mycobacterioses, especially TB.^17^ This vitamin has a remarkable effect on innate immunity, where it plays a pro-inflammatory role by activating macrophages via direct binding of 1,25(OH)_2_-vitamin D to the vitamin D receptor (VDR). VDR acts as a transcription factor in numerous immune cells when activated by the active form of vitamin D.^18^ ^,^ ^19^

Although vitamin D levels are not directly associated with leprosy, polymorphisms in the *VDR* gene have been correlated with disease progression and a higher bacilloscopic index.^20^ ^,^ ^21^ Some studies have associated polymorphisms of the *VDR* gene with the risk of leprosy in the Brazilian population and attributed them to the control of the Th1/Th2 cytokine balance, which predicts the clinical evolution of leprosy.^22^

Oliveira et al.^23^ suggest a correlation between serum IL-2 levels in untreated PB and MB leprosy patients and *VDR* and *CAMP* gene expression. IL-2 is a pro-inflammatory cytokine involved in the differentiation of T cells, and in PB patients it is involved in the differentiation of TCD4+ lymphocytes into Th1 or Th17. These authors also observed a strong correlation between *VDR* gene expression and IFN-γ levels in patients undergoing multidrug therapy (MDT). This cytokine is responsible for maintaining the inflammatory response in MB patients, as well as for inducing Th2, Th17, natural killer, B cells, macrophages, neutrophils, and eosinophils.^23^

Studies focusing on the influence of nutrients on leprosy are few and limited. Despite that, there is evidence for a strong association between leprosy and nutritional deficiencies.^15^ ^,^ ^24^ ^,^ ^25^ Most studies describe the nutritional profile of leprosy patients after diagnosis; however, the influence of nutritional status prior to infection on leprosy outcome is not understood.^7^ ^,^ ^15^ ^,^ ^24^ ^,^ ^26^ The multidirectional interaction between nutrition, infection, and immunity is an important aspect to consider in infectious diseases based on the clinical and immunological impact of micronutrient deficiency and supplementation.^27^ ^,^ ^28^ Therefore, determining a causal relationship between nutrition and leprosy is a challenge, and a better understanding of this topic can impact disease control, prevention, and intervention actions in the most affected populations.^26^

Thus, our study investigated the effect of iron or vitamin D dietary deficiency or supplementation on the inflammatory response of murine peritoneal cells and splenocytes from BALB/c mice stimulated *in vitro* with *M. leprae*.

## MATERIALS AND METHODS

*Study design* - The experimental design of this study was based on male BALB/c mice that received modified diets (Pragsolutions Biosciences, São Paulo, Brazil) immediately after weaning, offered ad libitum and maintained until euthanasia. Animals were housed in 12/12 light/dark room light cycles (lights turned on at 7:00 am and turn off at 7:00 pm). Mice were divided into five groups of five animals according to the following experimental diets:

Standard: standard adult maintenance mouse diet AIN-93M (minimum concentration of vitamin D of 2.000 IU/kg and 50ppm of iron);^29^

Iron-deficient: modified diet with 0% iron;

Iron-supplemented: modified diet with 500 ppm of iron;

Vitamin D-deficient: modified diet with 0 IU/kg of vitamin D;

Vitamin D-supplemented: modified diet with 10.000 IU/kg of vitamin D.

The animals received the diets ad libitum for 30 days and then were euthanised for blood collection, peritoneal cells and splenocytes harvest to perform the *in vitro* experiments.

*Serum iron and vitamin D levels* - Iron and 25(OH)-vitamin D were measured in the serum of mice collected by cardiac puncture after euthanasia. The quantification of serum iron was performed by colorimetric method using the Quimifer commercial kit (Ebram, São Paulo, SP, Brazil) with a detection range from 0 to 1000 µg/dL. The dosage of 25(OH)-vitamin D was performed by turbidimetry using the Turb Vit-D commercial kit (Ebram, São Paulo, SP, Brazil) with detection range of 7.6 to 147.8 ng/mL. Both levels were determined in the automated biochemical equipment BS-300 (Mindray, São Paulo, SP, Brazil).

*Hepatic iron deposit assessment* - The presence of iron in the livers of mice receiving standard, supplemented, and iron-deficient diets was evaluated using the Perls staining method. Briefly, the livers were fixed in formalin, embedded in paraffin, and cut into 5 µm-thick paraffin sections. The sections were deparaffinised at 60ºC followed by baths in xylene and hydrated in ethanol gradients (100%, 90%, and 70%) for staining. Sections were then incubated in the Perls’ staining solution (10% potassium ferrocyanide and 20% hydrochloric acid) for 30 min and, after washing with distilled water, were counterstained with nuclear fast red for 20 min. The slides underwent rapid ethanol gradient dehydration, fixed and mounted in an aqueous Glycergel medium (Agilent, Santa Clara, California, USA). The liver iron contents were observed under an optical microscope, and the representative images were collected.

*Culture of peritoneal cells* - Total peritoneal cells were obtained by washing the peritoneal cavity with phosphate-buffered saline (PBS) supplemented with 3% foetal bovine serum (FBS) followed by centrifugation at 1.500 rpm for 10 min at 4ºC. The supernatant was discarded and the cell pellet was resuspended in RPMI medium (Life Technologies, Carlsbad, California, United States) supplemented with 10% FBS and 1% antibiotic (penicillin 100U/mL and streptomycin A 100 µg/mL). The cell concentration was adjusted to 1 x 10^6^ cells/mL and 200 µL of the suspension was distributed per well in 24-well plates.

Cells were cultured in the presence of *M. leprae* for 24 h at 37ºC in a humidified atmosphere with 5% carbon dioxide (CO_2_) at a multiplicity of infection of 10:1. As a positive control, peritoneal cells were stimulated with *Escherichia coli* lipopolysaccharide (LPS) (Sigma-Aldrich, Saint Louis, Missouri, United States) at a final concentration of 10 µg/mL. As a negative control, peritoneal cells were cultured in an identical supplemented medium in the absence of stimulus. The supernatants were collected for measurements of cytokines, nitric oxide and hydrogen peroxide.

*Purification of M. leprae for infection of peritoneal cells* - The *M. leprae* used for *in vitro* cellular infection was obtained from the footpads of previously inoculated athymic nude mice with the Thai 53 strain of *M. leprae*, according to the protocol of Trombone et al.^30^. Briefly, the protocol for the recovery of bacilli included dissection of the footpad of mice, tissue isolation, and homogenisation (Turrax-IKA, North Carolina, United States) followed by purification by enzymatic digestion with 0.05% trypsin. Enumeration of *M. leprae* was performed after Ziehl-Neelsen staining by optical microscopy.

*Culture of splenocytes* - Splenic cells were obtained after maceration of spleens with RPMI medium and recovered by centrifugation at 1,500 rpm for 10 min at 4ºC. Supernatant was then discarded and the cells were resuspended in lysis buffer (0.15M NH_4_Cl) and incubated for 5 min at room temperature for osmotic lysis of the erythrocytes. Afterward, a washing step and inactivation of the lysis buffer with RPMI were carried out. The cells were immediately resuspended in RPMI supplemented with 10% FBS and 1% antibiotic (penicillin 100U/mL and streptomycin A 100 µg/mL), counted in a Neubauer chamber with Trypan blue (1:2 ratio) and the cell concentration was adjusted to 5 x 10^6^ cells/mL and 100 µL of the suspension was distributed into 96-well plates.

The cells were stimulated with sonicated *M. leprae* antigen at the final concentration of 10 µg/mL (kindly provided by Dr Patrick Brennan, Colorado State University, USA). As a positive control, splenocytes were stimulated with Concanavalin A (*ConA*) (Sigma-Aldrich, Saint Louis, Missouri, United States) at the final concentration of 2.5 µg/mL, and as a negative control splenocytes were cultured only with supplemented RPMI medium. The cultures were incubated for 72 h at 37ºC in a humid atmosphere with 5% CO_2_. Cultures supernatants were collected and stored at -80ºC for cytokine dosage. Splenocytes stimulated with sonicated *M. leprae* antigen, *ConA* and control culture were used for the assay to evaluate cell proliferation.

*Dosage of nitric oxide* - The nitric oxide (NO) dosage in the supernatants of the peritoneal cells culture was performed by the detection of nitrite (NO_2_) by the Griess colorimetric method.^31^ In synthesis, 100 µL of Griess reagent (0.1% N-Naphthyl-Ethylenediamine, 1% sulphanilamide, 0.5 mol L-1 H_3_PO_4_) were added to 100 µL of the culture supernatants in a 96-well plate and incubated for 10 min at room temperature. Absorbance was determined in an enzyme-linked immunosorbent assay (ELISA) reader (ThermoFisher Scientific, Waltham, Massachusetts, United States) at a wavelength of 540 nm. The NO_2_ concentration was determined by comparison with a standard curve constructed with sodium nitrite at concentrations from 0.19 to 200 µM. The dosages were made in duplicates.

*Dosage of hydrogen peroxide* - The dosage of hydrogen peroxide (H_2_O_2_) was performed in the culture of peritoneal cells stimulated with the *M. leprae* suspension, according to the Pick and Mizel method.^32^ For that 400 µL of phenol red buffer solution, and 40 µL of phorbol myristate acetate (PMA) were added to the culture and incubated at 37ºC in a humid atmosphere with 5% CO_2_ for 60 min. After incubation, 40 µL of 1 M sodium hydroxide was added and absorbance immediately read in an ELISA reader at 620 nm (ThermoFisher Scientific, Waltham, Massachusetts, United States). The H_2_O_2_ concentration was determined in nM by comparison with a standard curve constructed with concentrations of 0.5 to 2 nM. Dosages were made in triplicates.

*Evaluation of cell proliferation* - Cell proliferation of splenocyte culture was evaluated by the MTT assay (3-(4,5-dimethythiazole-2-yl)-2,5-diphenyltetrazolium bromide). After 72 h of cultivation, 20 µL of MTT (Sigma-Aldrich, Saint Louis, Missouri, United States) solution was added to the culture wells at 5 mg/mL with incubation of 2 h at 37ºC in a humid atmosphere with 5% CO_2_. The plates were then centrifuged at 1500 rpm for 10 min and the supernatants were carefully discarded. Then, 100 µL of DMSO (dimethyl sulfoxide, Sigma-Aldrich, Saint Louis, Missouri, United States) were added to each well. After 5 min, the plates were read at 540 nm in an ELISA reader (ThermoFisher Scientific, Waltham, Massachusetts, United States). The dosages were made in triplicates. The absorbances of the control cells of each diet were considered as 100% cell viability and used as a reference for the percentage calculations of the viability of the stimulated cells.

*Cytokine dosage by flow cytometry* - The supernatants of peritoneal cells and splenocytes cultures were quantified for Th1, Th2 and Th17 profile cytokine levels using the Cytometric Bead Array (CBA) Mouse Th1/Th2/Th17 CBA Kit (BD Biosciences, Franklin Lakes, New Jersey, United States) assay, according to the manufacturer’s instructions. Briefly, the cultures supernatants and cytokine patterns, previously diluted, were mixed with a pool of beads (IL-2, IL-4, IL-6, IL10, IL-17A, IFN-γ and TNF) and the detection reagent PE, and incubated for 120 min at room temperature, in the dark. After incubation, the capture spheres were washed twice with wash buffer, the supernatant carefully discarded and the beads resuspended in wash buffer for analysis in flow cytometry equipment FACSCalibur (BD Biosciences, Franklin Lakes, New Jersey, United States). After data collection, cytokine concentrations were calculated based on the results of the standard curves using the FCAP Array software (BD Biosciences, Franklin Lakes, New Jersey, United States).

*Ethics approval* - This study was approved by the Ethics Committee on Animal Use (CEUA-ILSL: 003/18) of the Lauro de Souza Lima Institute.

*Statistical analysis* - For the comparisons among standard, deficient, and supplemented diets, or different stimuli, we employed multiple comparison tests. The multiple comparison test establishing the standard diet group as a control was used to compare the effects of imbalance with the ideal condition. Furthermore, supplementation and deficiency conditions were compared through comparison tests between the two groups.

The data were analysed regarding the distribution model using the Shapiro-Wilk test. For data with normal distribution, Student’s t-test and/or ANOVA, followed by Tukey’s post-test for multiple comparisons, or Dunnett test for comparisons with the control group (standard diet), were used. For non-parametric data, the Mann-Whitney test and/or Kruskal-Wallis test followed by the Dunn test for multiple comparisons were used.

All statistical analyses were performed in GraphPad Prism version 9.0.0, adopting a significance level of p-value < 0.05.

## RESULTS

**Fig. 1:**
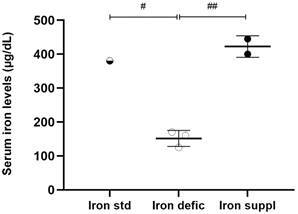
iron serum levels in mice after standard diet or with modification of iron content diet. Dosage performed by colorimetry. Values expressed in μg/dL. # p < 0.05 and ## p < 0.001 represents statistical significance by t-test. Iron std: standard iron diet; Iron defic: iron-deficient diet; Iron suppl: iron-supplemented diet.

*Serum iron and vitamin D levels and hepatic iron stores in mice after a modified diet* - Diets modified in iron content showed significantly different levels of this metal between the groups of this study, which validates the expected effects of these diets (Fig. 1).

The serum iron values obtained with the iron-deficient diet were significantly lower than the standard diet (p = 0,01) and iron-supplemented diet (p = 0,001). Serum iron levels between mice receiving the standard diet and mice receiving the iron-supplemented diet remained close and showed no statistical difference.

**Fig. 2:**
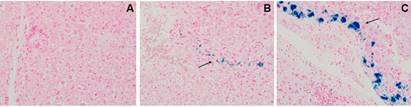
photomicrograph of histological section of liver from BALB/c mice that received a modified iron diet or standard diet. Representative liver sections stained with Perls Prussian blue for iron. (A) Mouse subjected to iron-deficient diet. (B) Mouse subjected to standard diet. (C) Mouse subjected to iron-supplemented diet. Black arrow indicates iron deposition. Magnification: 400 x.

Different hepatic iron accumulation resulting from the diets was evidenced by a strong positive reaction to iron with a pattern of multifocal accumulation in the tissue of livers from mice that received the iron-supplemented diet (Fig. 2C), while a weak and isolated deposition of iron was observed in the liver of mice fed with a standard diet (Fig. 2B), and no deposit in the liver from mice fed with the restricted diet (Fig. 2A).

Measurements of serum vitamin D were performed using pooled serum samples from each group. No vitamin D levels were detected in the groups that received standard or vitamin D-restricted diets. In contrast, the vitamin D dosage for the pool of three serum samples from supplemented mice was 18.3 ng/mL.

**Fig. 3:**
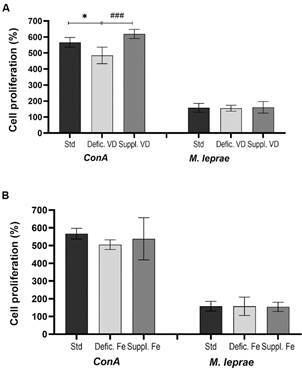
proliferation of splenocytes obtained from mice with different dietary conditions. Splenocytes were stimulated *in vitro* with *ConA* or sonicated *Mycobacterium leprae* antigen for 72 h. (A) Splenocytes recovered from mice on a modified vitamin D diet. (B) splenocytes recovered from mice on modified iron diet. The percentage of proliferation for each diet was calculated assuming the mean absorbance of splenocytes without stimulation as 100% cell viability. *p < 0.0001 represents the statistical significance by ANOVA; ### p < 0.0001 represents the statistical significance by t-test. Std: standard diet; Defic. VD: vitamin D deficient; Suppl. VD: vitamin D supplemented; Defic. Fe: iron deficient; Suppl. Fe: iron supplemented; *M. leprae*: sonicated *M. leprae* antigen; ConA: *Concanavalin A.*

*Vitamin D deficiency decreases Concanavalin A-induced splenocyte proliferation* - The modification of the vitamin D diet influenced the proliferative response of splenocytes *in vitro* only when stimulated by *ConA* (Fig. 3A). In this condition, vitamin D deficiency induced a significantly lower proliferative response of splenocytes when compared to the vitamin D-supplemented diet (p = 0.0009) and the standard diet (p = 0.01). However, no effect was observed when splenocytes were stimulated by *M. leprae* antigen*.*

Splenocytes obtained from mice with iron-supplemented or iron-deficient diets exhibited the same level of proliferative response of splenocytes from the standard diet when stimulated by *ConA* or sonicated *M. leprae* antigen (Fig. 3B).

The *in vitro* stimulation of peritoneal cells with *M. leprae* suspension did not induce the release of large amounts of H_2_O_2_, and no statistically significant differences were observed for any of the evaluated diets. The NO levels present in the supernatants of the peritoneal macrophage culture were not significantly different for any of the *in vitro* stimuli or diets evaluated (data not showed).

**Fig. 4:**
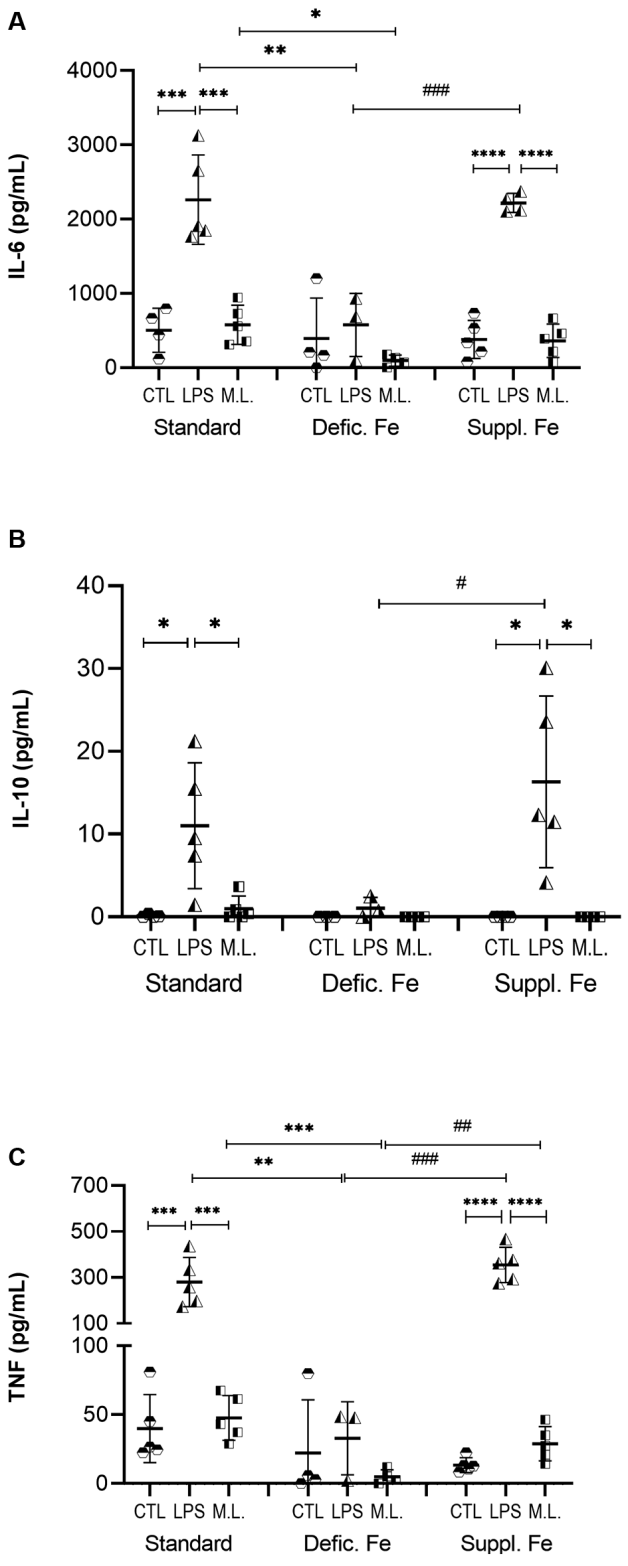
cytokine production by peritoneal cells from mice with different dietary iron conditions and stimulated *in vitro* with *Mycobacterium leprae*. Peritoneal cells were recovered from mice with a standard diet, iron-deficient diet, or iron-supplemented diet, and were stimulated with *M. leprae* or lipopolysaccharide (LPS), or non-stimulated. *p < 0.05; **p < 0.01; ***p < 0.001; ****p < 0.0001 represents statistical significance by analysis of variance (ANOVA). # p < 0.05; ## p < 0.01; ### p < 0.001 represents statistical significance by t-test. Defic. Fe: iron deficient; Suppl. Fe: iron supplemented; ML: *M. leprae*; CTL: control culture.

*Dietary iron restriction inhibits the pro-inflammatory response of peritoneal cells to M. leprae in vitro* - In our study, iron deficiency resulted in lower secretion of IL-6 and TNF by peritoneal cells infected *in vitro* with *M. leprae* (Fig. 4). In this condition, the production of IL-6 was significantly lower than the standard diet (p = 0.01). The secretion of TNF by peritoneal cells from iron deficiency stimulated with *M. leprae* was lower than the standard diet (p = 0.0007) and iron-supplemented diet (p = 0.009).

No significant levels of IL-10 were detected in the control and *M. leprae* stimulated cultures, and only low levels were detected in the presence of LPS. However, IL-10 levels with the LPS stimulus were significantly different between supplementation and deficiency diets (p = 0.049), and iron deficiency inhibited the secretion of this cytokine.

**Fig. 5:**
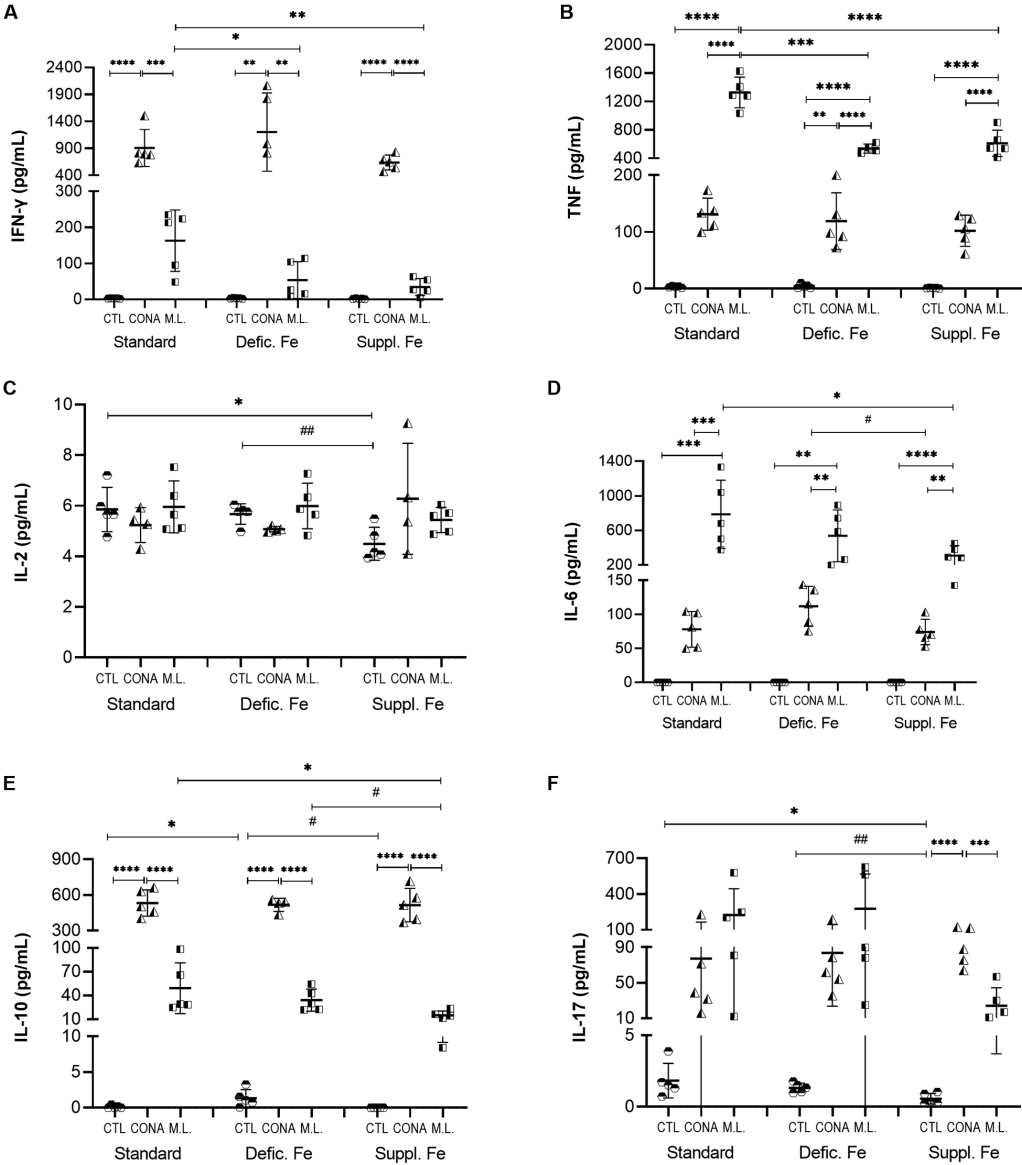
cytokine production by splenocytes from mice with different dietary iron conditions and stimulated *in vitro* with sonicated *Mycobacterium leprae* antigens. The splenocytes were recovered from mice with a standard diet, iron-deficient diet, or iron-supplemented diet. *p < 0.05; **p < 0.01; ***p < 0.001; ****p < 0.0001 represents statistical significance by analysis of variance (ANOVA). # p < 0.05; # p < 0.01 represents statistical significance by t-test. Defic. Fe: iron deficient; Suppl. Fe: iron supplemented; ML: sonicated *M. leprae* antigen; CTL: control culture; CONA: Concanavalin A.

*The inflammatory response of splenocytes to M. leprae antigens is modified by the iron content diet* - The production of IFN-γ and TNF by splenocytes stimulated with the sonicated *M. leprae* antigen *e* was significantly different among the diets evaluated (Fig. 5). There was a significant decrease in the production of IFN-γ in both iron supplementation and deficiency compared to the secretion level obtained from splenocytes of the standard diet (p = 0.009 and p = 0.02, respectively). This same reduction was observed for TNF when we compared the diets with variations of iron contents with the standard diet (p < 0.0001).

In cultures of splenocytes stimulated with *M. leprae*, we also observed that iron supplementation significantly reduced the production of IL-6 (p = 0.04) and IL-10 (p = 0.03) compared to the standard diet. The lower IL-10 production in the iron-supplemented diet group was also statistically different from the restricted diet group (p = 0.02).

The cultures of splenocytes stimulated with *M. leprae* presented a significantly higher production of IL-6 and TNF than in those stimulated with *ConA* for all the diets tested.

The production of IFN-γ, TNF, and IL-10 by splenocytes stimulated with *ConA* was not influenced by different dietary conditions. However, under *ConA* stimulus, the production of IL-6 was reduced in iron supplementation compared to the iron-deficient diet.

The production of IL-2 was low in all culture conditions or diets, and only for unstimulated cultures, we observed statistical differences. Under this condition, iron supplementation decreased the secretion of IL-2 when compared to the standard diet (p = 0.01) and iron-deficient diet (p = 0.009).

Regarding the levels of IL-17A secretion in the control culture, splenocytes from animals that received a diet supplemented with iron produced lower levels of IL-17A when compared to the standard diet (p = 0.03) or the iron-deficient diet (p = 0.009).

IL-4 production occurred only when splenocytes were stimulated with *ConA*, and no statistical differences were detected among the different diets (data not shown).

**Fig. 6:**
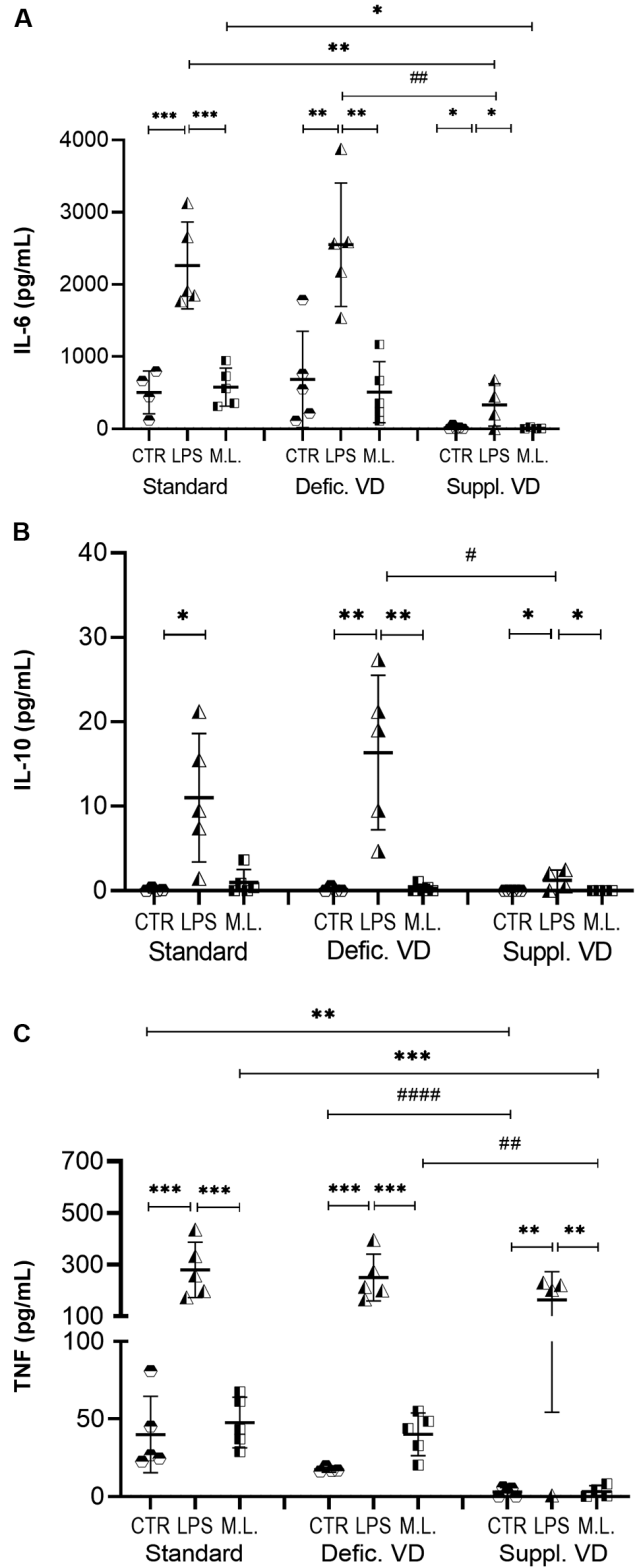
cytokine production by peritoneal cells from mice with different dietary conditions of vitamin D and stimulated *in vitro* with *Mycobacterium leprae*. Peritoneal cells were recovered from mice with a standard diet, vitamin D-deficient diet, or vitamin D-supplemented diet and were stimulated with *M. leprae* or LPS, or non-stimulated. *p < 0.05; **p < 0.01; ***p < 0.001 represents statistical significance by analysis of variance (ANOVA) or Kruskal-Wallis. # p < 0.05; # p < 0.01; #### p < 0.0001 represents statistical significance by t-test or Mann-Whitney. Defic. VD: vitamin D deficiency; Suppl. VD: supplemented with vitamin D; ML: *M. leprae*; CTL: control culture; LPS: lipopolysaccharide.

*High levels of vitamin D reduce pro-inflammatory cytokines and IL-10 production from peritoneal cells stimulated in vitro with M. leprae* - Vitamin D supplementation significantly reduced the secretion of IL-6 by peritoneal cell stimulated with *M. leprae* compared to the standard diet (p *=* 0.02) (Fig. 6). This same profile was observed for the stimulus with LPS compared to vitamin D-deficient (p = 0.001) and standard diets (p = 0.001).

The TNF production in peritoneal cells stimulated with *M. leprae* was also lower in the vitamin D supplementation condition compared to the standard diet (p = 0.0007) and vitamin D deficiency (p < 0.001).

The production of IL-10 by peritoneal cells was detected only in cultures stimulated with LPS, where vitamin D supplementation also exhibited a reducing effect on the production of this cytokine (p = 0.01) compared to vitamin D restriction.

**Fig. 7:**
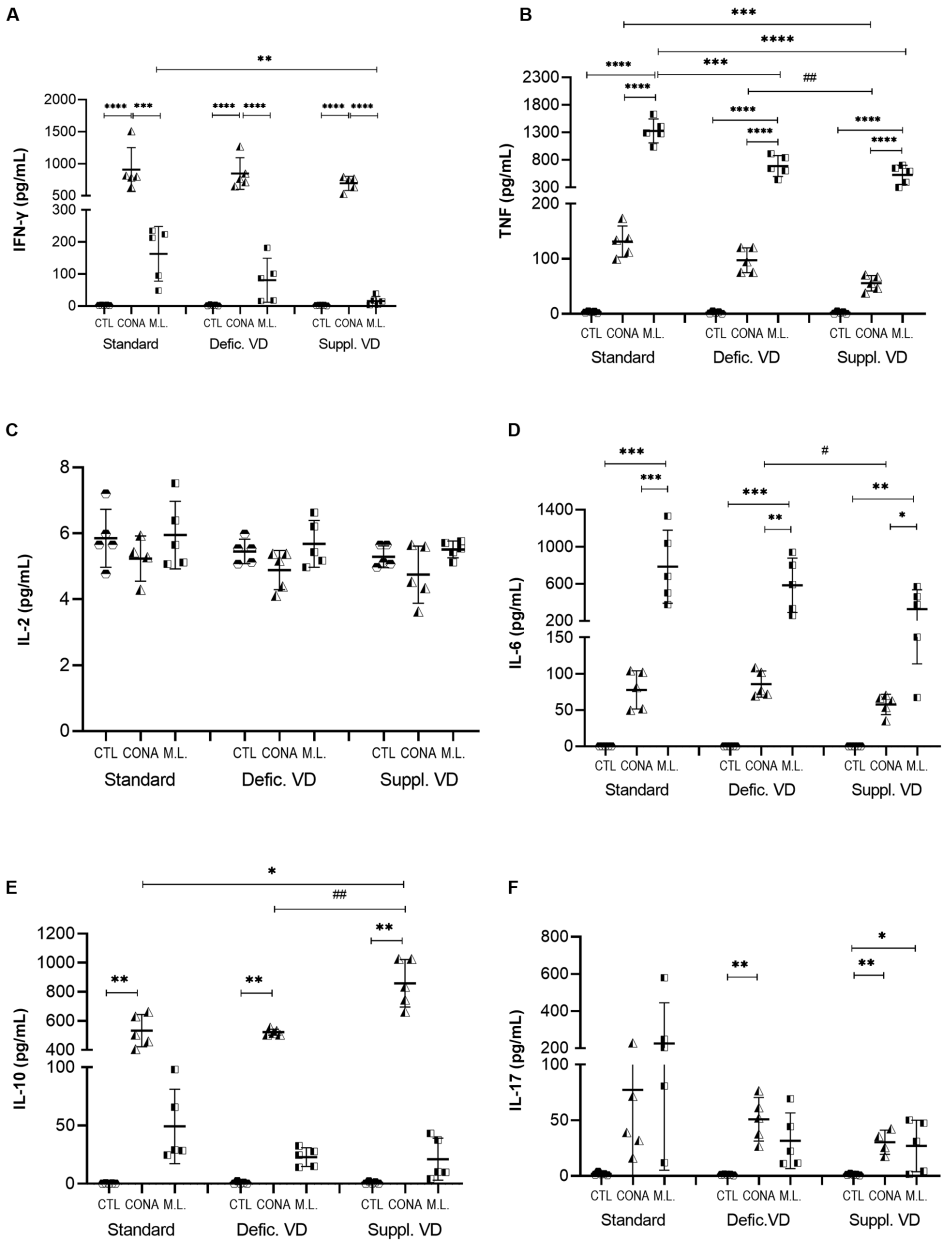
cytokine production by splenocytes from mice with different dietary conditions of vitamin D and stimulated *in vitro* with sonicated *Mycobacterium leprae* antigens. The splenocytes were recovered from mice with a standard diet, vitamin D-deficient diet, or vitamin D-supplemented diet. *p < 0.05; **p < 0.01; ***p < 0.001; ****p < 0.0001 represents statistical significance by analysis of variance (ANOVA) or Kruskal-Wallis. # p < 0.05; ## p < 0.01 represents statistical significance by t-test or Mann-Whitney. Defic. VD: vitamin D deficiency; Suppl. VD: supplemented with vitamin D; ML: sonicated *M. leprae* antigen; CTL: control culture; CONA: Concanavalin A.

*Anti-inflammatory effect of vitamin D in splenocytes stimulated with Mycobacterium leprae* - In general, we observed decreased cytokine production by splenocytes from mice subjected to *in vivo* vitamin D supplementation or restriction, when stimulated *in vitro* with *ConA* or *M. leprae* (Fig. 7).

Vitamin D supplementation significantly decreased the production of IFN-γ and TNF in splenocyte cultures stimulated with the sonicated *M. leprae* antigen compared to the standard diet (p = 0.006 and p < 0.0001, respectively). For TNF, this reduction was also observed for a restricted diet in relation to the standard diet (p = 0.0004).

The levels of IL-10 secretion for the stimulus with *ConA* were significantly higher in the vitamin D-supplemented diet in relation to the standard diet (p = 0,01) and vitamin D-deficient diet (p = 0,007) (Fig. 7F).

No differences were observed in the production of IL-17A for the stimulus with *M. leprae* among the different diets.

The production of IL-2 by splenocytes was low in all culture conditions and showed no statistical differences (Fig. 7C).

## DISCUSSION

The increasing relevance of understanding the effects of nutritional status on the outcome of infectious diseases is evidenced by the relevant associations of iron and vitamin D metabolism with disease progression and the host immune response.^33^ ^,^ ^34^ ^,^ ^35^ It is vital to note that this concept might be more applicable in the context of diseases affected by social determinants of health, such as leprosy.

Our results reveal that murine peritoneal cells recovered from animals subjected to a vitamin D-supplemented diet and activated with *M. leprae* produced low levels of the pro-inflammatory cytokines TNF and IL-6. Korf et al.^36^ discovered that the preconditioning macrophages with the active form of vitamin D alters their inflammatory profile and interferes with the production of cytokines and chemokines. These authors noticed a reduction in inflammatory mediators IL-12, TNF, and nitric oxide synthase, as well as low mRNA expression of the T cell recruiter chemokines CXCL9, CXCL10 and CXCL1, when they primed murine peritoneal cells *in vitro* with 1.25(OH)D before inducing an inflammatory response with LPS.^36^ Zhang et al.^37^ reported the same reduction when they pre-treated human monocytes with 1.25(OH)-vitamin D and 25(OH)-vitamin D. Harishankar et al.^38^ reported an inhibition effect on cytokines IL-1α, TNF, LT-α, IL-17 and IL-23 mediated by 1.25(OH)-vitamin D in the presence of *MTB* antigens in peripheral blood mononuclear cell culture of healthy controls and patients with pulmonary TB. These authors speculate that different inhibitory mechanisms, such as toll-like receptors and increased production of IL-10, may be involved in the negative regulation of pro-inflammatory cytokines by 1.25(OH)-vitamin D, with consequent decreased expression of Th1 and Th17 cytokines.^38^ Thus, our vitamin D supplementation *in vivo* model presented the same inhibitory effects as those found *in vitro*. The peritoneal cells derived from the iron-deficient diet mice showed reduced production of the pro-inflammatory cytokines IL-6 and TNF. It is well-documented that disruption of iron homeostasis can have several effects on immunological responses.^39^ The macrophages are involved in iron homeostasis, and intracellular iron storage influences the polarisation of these phagocytes and, consequently, the inflammatory response.^40^ ^,^ ^41^ Wang et al.^41^ also correlated low intracellular iron in macrophages to a decrease in the production of pro-inflammatory cytokines, such as IL-6 and TNF. This data supports our findings, since we confirmed low iron content in the serum and liver of mice subject to an iron-restricted diet.

Interestingly, all iron-modified diets evaluated decreased IFN-γ and TNF production by splenocytes after stimulation with *M. leprae* antigens in relation to a standard diet. The decrease in these pro-inflammatory cytokines in iron restriction is consistent with the inflammatory response suppression profile mediated by nutritional deficiency.^42^ ^,^ ^43^ ^,^ ^44^ Besides, some authors have already described the suppressive effects of iron overload on IFN-γ and TNF production by splenocytes.^45^ ^,^ ^46^ ^,^ ^47^ In the same trend, iron supplementation decreased the production of IL-6 and IL-10 by splenocytes stimulated with *M. leprae* antigens. The reducing effect on the production of IL-6 under iron supplementation has been described in other studies.^48^ Data concerning IL-10 production by splenocytes under different iron availabilities are not described in the literature. These findings reinforce the notion that optimal iron concentrations are critical for immune function balance.

In adaptive immunity, vitamin D is a metabolite that does not exert an effect on naive T cells, but exhibits distinct actions in differentiated subpopulations of T cells. The active form of vitamin D can suppress the production of IFN-γ and IL-2 in Th1, IL-4 in Th2, and IL17A in Th17, and positively regulate the differentiation of regulatory T cells that mediate the suppression of macrophage activity and produce IL-10 and TGF-β.^49^ ^,^ ^50^ Yang et al.^17^ observed that supplementation with vitamin D *in vivo* increased IL-10 and decreased IFN-γ production in splenic murine lymphocytes stimulated with tuberculin. This modulation seems to correspond with our results since splenocytes recovered from mice fed with the diet with a high concentration of vitamin D exhibited lower production of IFN-γ.

Vitamin D is a positive regulator of the synthesis of IL-10, a cytokine significantly related to immunological tolerance.^51^ The higher production of IL-10 in the culture of splenocytes stimulated with *ConA* after supplementation with vitamin D validates the anti-inflammatory character of this vitamin observed in our study. The most noticeable effect of vitamin D is on innate immunity, where it promotes inflammation by activating macrophages via 25(OH)-vitamin D binding to VDR. However, a variety of pro- and anti-inflammatory effects of vitamin D have been reported, and in particular, studies related to inflammation and autoimmunity show the anti-inflammatory activity of vitamin D, mediated mainly by its active form (1,25(OH)D), which reduces the production of pro-inflammatory cytokines.^49^ ^,^ ^52^ There is no standardised model on the optimal vitamin D levels that can influence the balance of pro-inflammatory and anti-inflammatory cytokines during an active and acute responses.^17^ ^,^ ^38^ ^,^ ^53^

Unlike other studies, we did not evaluate the inflammatory responses of cells under supplementation or deficiency produced *in vitro*. We investigated the *in vitro* production of cytokines by immune cells from animals subjected to iron or vitamin D restriction or supplementation from diet. However, we believe that our results point to the effectiveness of this model in simulating the effect of individual nutritional status on cellular responsiveness and disease outcome. However, one limitation of this study is that we did not perform phenotypic characterisation of the cells isolated from the peritoneum and spleen. As a result, we cannot confirm whether different diets affected the composition of these populations.

The low detection rate of vitamin D in serum samples was another limiting factor of this study. The vitamin D levels found by other authors in the restricted diets are below the detection limit of the assay used, which may explain our results for this group.^54^ Regarding vitamin D supplementation, based on the differences in the production of cytokines for this dietary condition, as well as only for this diet it was possible to detect vitamin D levels, we can assume that the supplementation was able to increase the serum levels of vitamin D. It is important to mention that there are no validated data on the adequate level of vitamin D in murine diet formulations to induce deficiency or supplementation.^55^

Our results demonstrate that iron and vitamin D levels interfere with the interaction between the host and *M. leprae* and, hence, have the potential to interfere with the progression of leprosy. The data obtained highlight the impact of nutritional aspects on this neglected disease, which is strongly linked to unfavoured social settings.
